# Pulmonary embolism in patients with chronic coronary syndrome masquerading as acute coronary syndrome: a case report and literature review

**DOI:** 10.1186/s12872-024-03998-6

**Published:** 2024-07-01

**Authors:** Yun-Hu Chen, Xing-Yu Zhu, Li-Hua Fan, Hong-Feng Xu

**Affiliations:** 1 Cardiovascular Department, Taicang TCM Hospital Affiliated to Nanjing University of Chinese Medicine, Suzhou, 215400 China; 2 Clinical Pharmacy Department, Taicang TCM Hospital Affiliated to Nanjing University of Chinese Medicine, Suzhou, 215400 China

**Keywords:** Pulmonary embolism, Syncope, D-dimer, Electrocardiogram, Case report

## Abstract

**Background:**

Pulmonary embolisms (PEs) exhibit clinical features similar to those of acute coronary syndrome (ACS), including electrocardiographic abnormalities and elevated troponin levels, which frequently lead to misdiagnoses in emergency situations.

**Case presentation:**

Here, we report a case of PE coinciding with chronic coronary syndrome in which the patient’s condition was obscured by symptoms mimicking ACS. A 68-year-old female with syncope presented to the hospital. Upon admission, she was found to have elevated troponin levels and an electrocardiogram showing ST-segment changes across multiple leads, which initially led to a diagnosis of ACS. Emergency coronary arteriography revealed occlusion of the posterior branches of the left ventricle of the right coronary artery, but based on the complexity of the intervention, the occlusion was considered chronic rather than acute. On the 3rd day after admission, the patient experienced recurrent chest tightness and shortness of breath, which was confirmed as acute PE by emergency computed tomography pulmonary angiography. Following standardized anticoagulation treatment, the patient improved and was subsequently discharged.

**Conclusions:**

This case report highlights the importance of recognizing the nonspecific features of PE. Clinicians should be vigilant when identifying other clinical features that are difficult to explain accompanying the expected disease, and it is necessary to carefully identify the causes to prevent missed diagnoses or misdiagnoses.

**Supplementary Information:**

The online version contains supplementary material available at 10.1186/s12872-024-03998-6.

## Background

Pulmonary embolism (PE) is the third leading cause of cardiovascular death following heart attack and stroke [1]. The clinical manifestations of PE can vary and include syncope, chest pain, dyspnea, and even sudden death. Data show that 70–80% of patients with acute PE present with abnormal electrocardiograms (ECGs), and 38.6% have elevated troponin levels [2, 3]. Acute PE shares similar clinical features with acute coronary syndrome (ACS) and can easily be misdiagnosed in emergency situations [4]. However, it should be noted that cases of simultaneous occurrence of PE and ACS have also been occasionally reported. Clinicians must maintain a high degree of suspicion to not only accurately distinguish PE from ACS but also to recognize the differences between ACS and chronic coronary syndrome (CCS). This information is essential for choosing the appropriate treatment strategy and reducing mortality. Here, we report a case of PE with CCS in which the true condition was masked by the illusion of ACS. Additionally, we provide a brief review of the relevant literature.

## Case presentation

A 68-year-old Chinese woman presented with a complaint of syncope that occurred 1 h earlier. One hour earlier, the patient experienced retrosternal stuffiness and discomfort while walking. Subsequently, she lost consciousness and fell, resulting in contusion of the forehead and soft tissue on the right side of the face. A few minutes later, the patient awoke spontaneously. Passers-by took her to the chest pain center at the hospital. The patient had a history of hypertension.

**Fig. 1:**
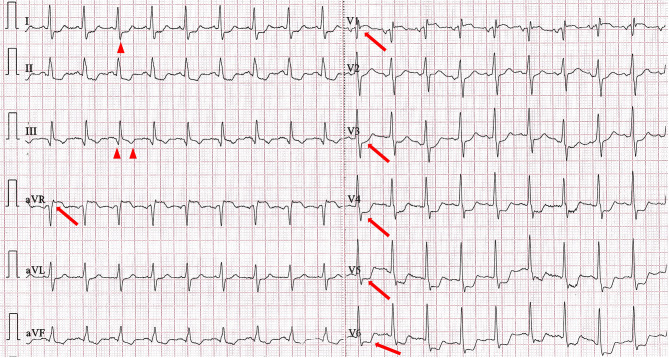
The patient’s first electrocardiogram recorded upon admission. ECG showed sinus tachycardia with a heart rate of 112 beats per minute, ST-segment elevation of 0.1–0.2 mV in leads AVR and V1, and ST-segment depression of 0.1–0.3 mV in leads V3-V6. This ECG has typical S1Q3T3 sign. Red arrows point to ST-segment alterations, and red triangles point to S1Q3T3 sign. ECG = Electrocardiogram

Her vital signs on admission were as follows: blood pressure 100/74 mmHg, heart rate 116 beats/min, respiration 22 breaths/min, and pulse oximetry SpO_2_ 96% (3 L/min oxygen inhalation). There was a 3 cm × 3 cm subcutaneous hematoma on the forehead with slight bleeding through the skin and a skin abrasion on the right side of the face. Cardiac examination revealed tachycardia and no valvular murmur, and neurological examination was negative. Blood tests revealed a serum troponin I concentration of 1.1 ng/ml (normal range: 0.01–0.023 ng/ml) and a D-dimer concentration of 14,400 ng/ml (normal range: 80–500 ng/ml). There were no obvious abnormalities in routine blood tests, liver or kidney function tests, coagulation function tests, or electrolyte tests. ECG revealed sinus tachycardia with a heart rate of 112 beats/minute, ST-segment elevation of 0.1–0.2 mV in leads AVR and V1, and ST-segment depression of 0.1–0.3 mV in leads V3-V6 (Fig. 1). The head and chest computed tomography scans showed no abnormalities.

**Fig. 2:**
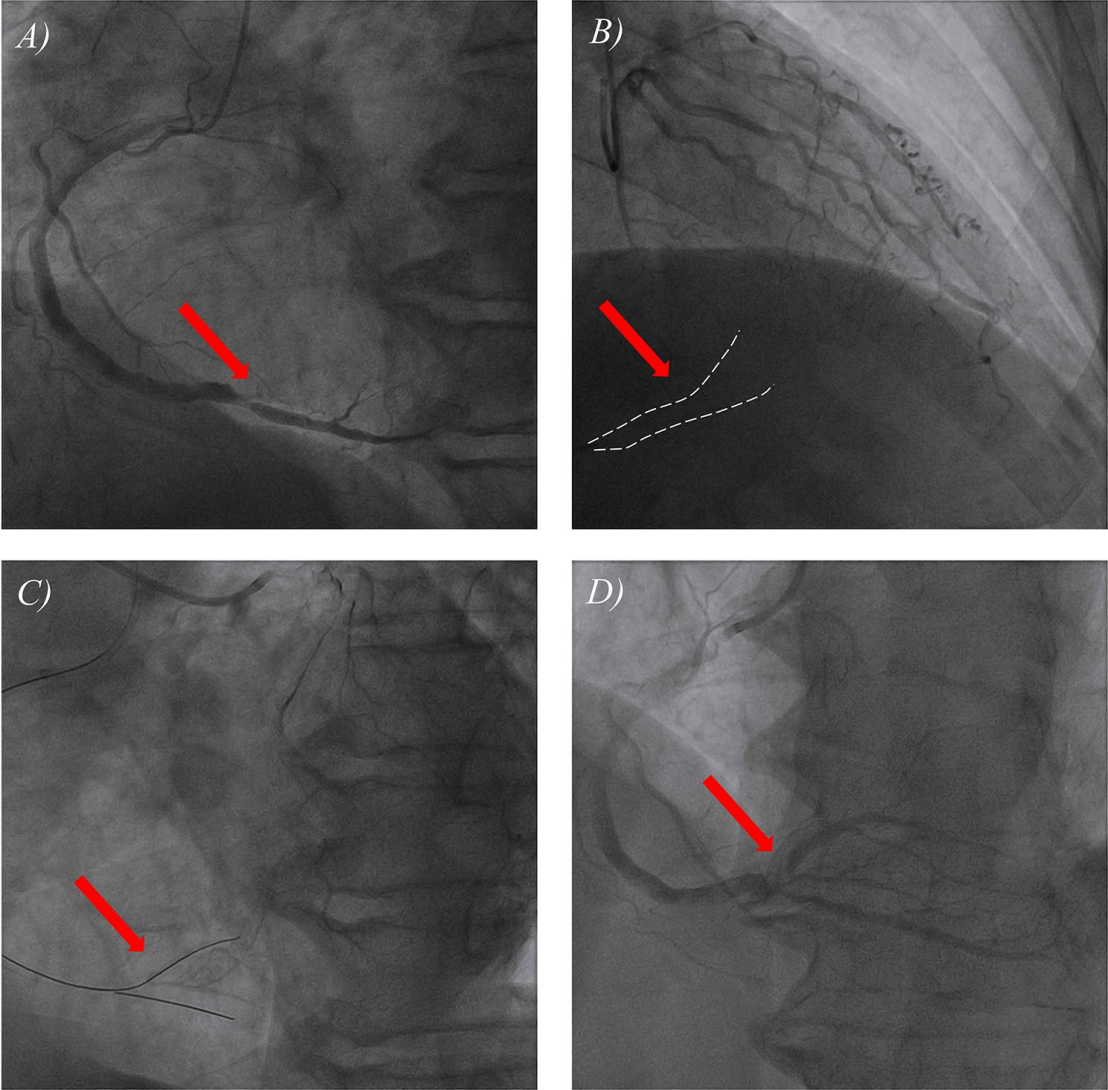
CAG images of the RCA for the patient. Panel A shows the initial CAG image of the RCA, with the red arrow indicating the onset of the occlusion in the vessel. In Panel B, the red arrow highlights the retrograde blood flow within the occluded segment, while the white dashed line outlines the flow’s profile. Panel C displays contralateral angiography confirming the guidewire’s placement within the true lumen of the vessel. Panel D presents the final CAG image of the RCA. The 4 videos corresponding to these figures can be found in the Supplementary materials. CAG = Coronary angiography, RCA = Right coronary artery

The patient was initially diagnosed with high-risk ACS. She was immediately administered 300 mg of aspirin, 180 mg of ticagrelor, 20 mg of atorvastatin, and a 3000 U intravenous bolus of heparin. Urgent coronary angiography (CAG) and revascularization were recommended. CAG revealed complete occlusion of the posterior branches of the left ventricle (PL) of the right coronary artery (RCA); thrombolysis in myocardial infarction (TIMI) grade 0 flow; no significant stenosis in the left main stem coronary artery (LM), left anterior descending artery (LAD), or left circumflex artery; and reverse blood flow in the distal LAD to the PLs (Fig. 2A and B and Supplementary Video 1, 2). In general, acute total occlusion (ATO) lesions in culprit vessels can be easily passed through using a working guidewire with a soft tip. However, the occlusion stiffness in this patient was much greater than expected and similar to that of patients with chronic total occlusion (CTO). After the 6 F SAL 1.0 guide catheter was inserted into the RCA opening, we first repeatedly attempted to use the Runthrough NS and ASAHI SION working guidewires but failed to pass through the occluded segment. A Fielder XT-R guidewire with a stiff tip was then used, but it also failed to pass through the occlusion. The surgical strategy was changed again, and a double-lumen microcatheter was used to deliver the Runthrough NS guidewire to the distal part of the posterior descending branch. After repeated rotation, the Gaia Second guidewire was passed through the occluded segment. Contralateral angiography confirmed that the guidewire was located in the true lumen of the vessel (Fig. 2C, Supplementary Video 3). The PL occlusion segment was dilated with a Maverick 1.5 mm×1.5 mm balloon (6–8 ATM ×10 s) and a Maverick 2.0 mm×1.5 mm balloon (8–10 ATM ×10 s). Repeat CAG showed approximately 80% stenosis of the PL opening, and TIMI flow was restored to grade 3 (Fig. 2D, Supplementary Video 4).

**Fig. 3:**
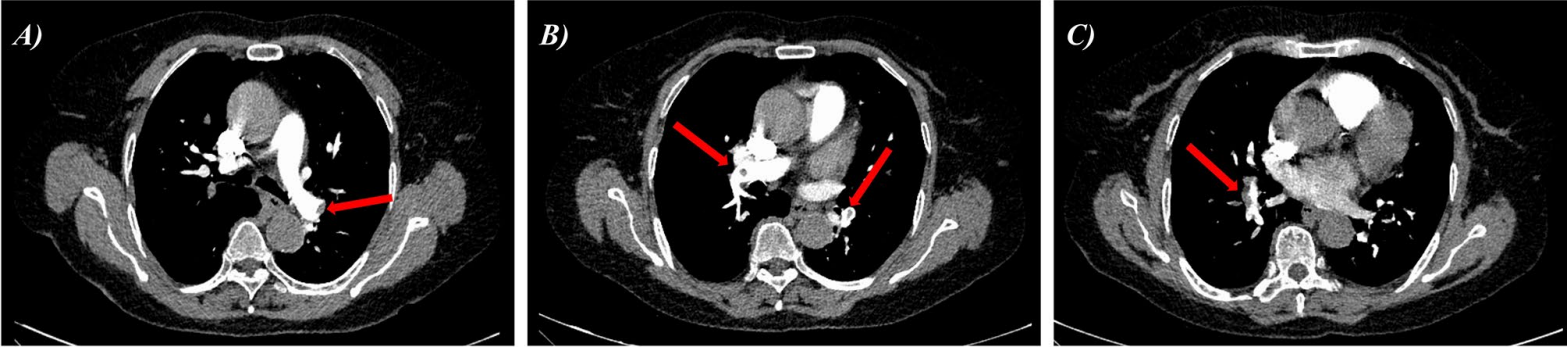
CTPA images showing pulmonary artery thrombi. The red arrows in Panels **A**, **B**, and **C** indicate thrombi at various levels extending to the distal end of the pulmonary artery. CTPA = computed tomography pulmonary angiography

On the next postoperative day, the patient reported relief of chest tightness and continued to take aspirin 100 mg qd, ticagrelor 90 mg bid, atorvastatin 20 mg qn, etc. Considering the patient’s frontal hematoma, anticoagulation therapy was not given because of the risk of worsening the hematoma. However, on the third day after admission, the patient developed chest tightness again, accompanied by dyspnea, and her D-dimer level reached 17,700 ng/ml. Urgent emergency computed tomography pulmonary angiography (CTPA) revealed massive thrombi in both the left and right pulmonary arteries (Fig. 3). Bedside echocardiography revealed right heart dilatation (right atrial transverse diameter, 42 mm; right ventricular transverse diameter, 38 mm), mildly elevated pulmonary artery pressure (38 mmHg), no thrombus in the ventricles, and a normal ejection fraction (61%). The risk stratification was medium risk, and an oral anticoagulant (rivaroxaban, 15 mg bid) was administered. We further screened for genetic thrombophilia, antiphospholipid syndrome, nephrotic syndrome, malignant tumors, lower extremity venous thrombosis, and other diseases to identify risk factors, but no positive findings were detected.

Ultimately, the patient was diagnosed with acute PE. During CAG, an occlusion in the PL was identified. Given the technical challenges associated with the procedure, this occlusion was more likely attributed to CTO within the context of CCS rather than ACS due to ATO. Two weeks later, the patient was discharged without any complications. After 6 months of follow-up, the patient was in good condition, and CTPA and echocardiography returned to normal.

## Discussion

We present a case of acute PE initially misdiagnosed as ACS. Initially, the focus on ACS obscured the correct diagnosis. A subsequent review of the clinical features and treatment responses revealed inconsistencies with ACS, emphasizing the necessity of prompt and precise diagnosis of PE to prevent catastrophic outcomes.

Syncope may be the primary or sole symptom of PE and is linked to a poor prognosis. The prevalence of PE among patients with syncope varies widely, with studies reporting rates ranging from 0.6–17.3% [5–8]. Likewise, the incidence of syncope among patients with PE ranges from 6–14.8% [9, 10]. Patients with PE who present with syncope are considered at high risk, even if syncope alone does not meet the criteria for high-risk PE. Syncope is prevalent among patients with high-risk PE, often due to right ventricular dysfunction. When ECG reveals ST-segment elevation in the anterior wall leads (V1-V4), the incidence of syncope in PE patients may reach 66.7% [2]. In PE patients, the presence of syncope is associated with an increased risk of experiencing major bleeding events. One study revealed that during the first 90 days of antithrombotic therapy, PE patients with syncope face a significantly greater risk of experiencing major bleeding than those without syncope [10]. In addition, patients with PE who experience syncope tend to be older and more susceptible to critical conditions, such as hemodynamic instability and elevated troponin levels, than those without syncope [10]. They also face a greater risk of fatal outcomes and in-hospital death. Consequently, PE patients with syncope are considered a high-risk group and are more vulnerable to complications and adverse outcomes, necessitating increased medical attention. Syncope may also occur in ACS patients with malignant arrhythmias or severe hypotension. The literature reports that approximately 14% of ACS patients may experience dizziness, presyncope, or syncope [11]. This undoubtedly adds to the confusion between PE and ACS, necessitating a more comprehensive diagnostic evaluation, including physical examination, ECG, troponin concentrations, and D-dimer concentrations, among others.

Although the use of ECG for detecting PE lacks specificity, it has been extensively studied. The ECGs of PE patients show rhythm and QRS-T wave changes [12]. The most common finding, sinus tachycardia, is generally attributed to a compensatory increase in heart rate following ischemia and hypoxia. This finding aligns with our reported case but contrasts with the patient’s CAG findings, as RCA occlusion typically results in significant bradycardia. The S1Q3T3 sign, a classic and relatively specific ECG pattern, occurs in only 15–25% of PE patients and has low sensitivity [13, 14]. The initial ECG of our patient displayed a significant S1Q3T3 sign, which unfortunately did not receive sufficient attention. Right bundle branch block is also commonly observed in the PE patient population. There is growing interest in ST-segment changes among PE patients. Since Falterman reported the first case of PE with ST-segment elevation in 2001, dozens of similar cases have emerged [15]. The ST-segment changes in PE primarily occur along the anterior septum, and the ECGs typically show ST-segment elevation or depression in the chest leads. Due to the significant dilation of the right ventricle caused by pulmonary hypertension, leads V1-V3, and occasionally V4, are positioned in front of the right ventricle’s anterior wall. Ultimately, this mismatch between oxygen supply and demand can lead to transmural ischemia or infarction of the right ventricle. Patients with PE may also exhibit ST-segment elevation in the lead aVR. A retrospective analysis revealed that 34.3% of patients with PE showed ST-segment elevation in the aVR, which must be differentiated from LM or 3-vessel ACS [16]. Furthermore, reports in the literature indicate that ST-segment elevation in patients with PE can also result from coronary artery embolism secondary to a patent foramen ovale; in such cases, both PE and coronary embolism coexist, leading to ECG changes that reflect the combined effects of these two conditions [17, 18]. Certain ECG features associated with PE can predict a worse prognosis. ST-segment elevations in leads V1 and aVR, along with ST-segment depressions in leads V4-V6, are recognized as predictors of cardiogenic shock and are linked to increased mortality. These features are highly consistent with our case and suggest that any delay in treatment is likely to result in cardiogenic shock or even death.

Several cardiac biomarkers are widely used for diagnosing and evaluating PE. The value of D-dimer concentrations in the context of PE has been confirmed through clinical practice, and D-dimer concentrations are considered one of the important criteria for diagnosing this condition. Although D-dimer is highly sensitive, it has very low specificity and may be elevated in various other conditions, such as old age, inflammation, hemorrhage, trauma, and cancer. This lack of specificity makes it difficult to reliably use D-dimer concentrations to diagnose many thrombotic disorders. However, a normal D-dimer level—defined as a cutoff of 500 µg/L in patients aged 50 or younger or an age-adjusted cutoff of (age × 10) µg/L in patients older than 50—often excludes PE, given that its negative predictive value is between 97% and 100% [6, 19, 20]. D-dimer levels were also associated with disease severity but not with long-term prognosis in acute PE patients. The role of cardiac biomarkers, such as troponin and brain natriuretic peptide (BNP) concentrations, in PE is also under intensive investigation. Troponin is a marker of myocardial injury, and PE may also lead to elevated troponin levels, especially in patients with right ventricular dysfunction. The role of troponin concentrations in identifying individuals at high risk for PE has been well evaluated, and elevated levels are strongly associated with short-term mortality and adverse outcome events [21]. Given that increases in both D-dimer and troponin concentrations can occur in patients with PE and those with ACS, some groups have suggested using the ratio of D-dimer to troponin to distinguish PE with increased troponin concentrations from acute non-ST-segment elevation myocardial infarction. Optimal cardiovascular imaging to identify PE is recommended for patients with a ratio greater than 1.82 (sensitivity 93.3%, specificity 86.6%) [3]. BNP or N-terminal proBNP (NT-proBNP) concentrations are another biomarker for risk stratification of adverse events in patients with acute PE. Negative levels of BNP or NT-proBNP can identify patients with PE who are at low risk and suitable for outpatient management, whereas patients with abnormal levels have a six- to sevenfold greater risk of death and adverse outcomes than patients with normal levels [22].

CTPA and echocardiography are the most important cardiovascular imaging methods for detecting pulmonary embolism. The widespread availability of CTPA has made it the most commonly used primary diagnostic tool for pulmonary embolism. However, CTPA is associated with potential risks such as radiation exposure and contrast agent reactions. Compared with CTPA, bedside echocardiography is a safe and highly valuable diagnostic and evaluation tool, especially for hemodynamically unstable patients with suspected pulmonary embolism who cannot undergo CTPA immediately. In such cases, echocardiography may play a key role, helping to avoid diagnostic pitfalls and unnecessary treatments. Elevated right ventricular pressure, right ventricular dilatation, and abnormal right ventricular activity were the most common echocardiographic findings. In patients with shock or hypotension, echocardiography is also a useful tool with high diagnostic predictive value.

Anticoagulant therapy is the cornerstone of PE treatment, and it is also an important treatment method for ACS. Overestimating the risk of bleeding caused by anticoagulant therapy may cause fatal harm. Our reported patient was initially diagnosed with ACS and was not treated with anticoagulation after surgery because the medical team overestimated the patient’s bleeding risk (subcutaneous hematoma), which undoubtedly objectively caused a delay in the treatment of PE. Indeed, contraindications to antithrombotic therapy are not uncommon in thrombotic disorders. A national cohort study in the U.S. showed that antithrombotic therapy was contraindicated for up to 18% of patients who underwent percutaneous coronary intervention [23]. Variability in the clinical characteristics of patients results in different bleeding risks associated with antithrombotic therapy. The need to find the optimal balance between thrombosis and bleeding risk highlights the need to develop and apply robust bleeding risk assessment tools aimed at optimizing ischemic protection while reducing bleeding risk.

## Conclusions

We report a case of pulmonary embolism with CCS that was initially misdiagnosed as ACS, resulting in delayed treatment. The clinical features of pulmonary embolism differ from those of ACS. Clinicians should be vigilant when identifying other clinical features that are difficult to explain accompanying the disease, and it is necessary to carefully identify the causes to prevent missed diagnoses or misdiagnoses.

### Electronic supplementary material

Below is the link to the electronic supplementary material.

Supplementary Video 1: CAG revealed complete occlusion of the PL of RCA, with TIMI grade 0 flow.

Supplementary Video 2: CAG revealed no significant stenosis in the LM, LAD, or left circumflex artery, and showed reverse blood flow from the distal LAD to the PLs.

Supplementary Video 3: Contralateral angiography confirmed that the guidewire was located in the true lumen of the vessel.

Supplementary Video 4: Repeat CAG showed approximately 80% stenosis of the PL opening, and TIMI flow was restored to grade 3.

## Data Availability

No datasets were generated or analysed during the current study.

## Abbreviations

PE: Pulmonary embolism
ACS: Acute coronary syndrome
ECG: Electrocardiogram
CAG: Coronary angiography
CCS: Chronic coronary syndrome
PL: Posterior branch of the left ventricle
RCA: Right coronary artery
TIMI: Thrombolysis in myocardial infarction
LM: Left main stem coronary artery
LAD: Left anterior descending artery
ATO: Acute total occlusion
CTO: Chronic total occlusion
CTPA: Computed tomography pulmonary angiography
BNP: Brain natriuretic peptide
NT-proBNP: N-terminal proBNP
